# Surface-Modified Poly(l-lactide-*co*-glycolide) Scaffolds for the Treatment of Osteochondral Critical Size Defects—In Vivo Studies on Rabbits

**DOI:** 10.3390/ijms21207541

**Published:** 2020-10-13

**Authors:** Małgorzata Krok-Borkowicz, Katarzyna Reczyńska, Łucja Rumian, Elżbieta Menaszek, Maciej Orzelski, Piotr Malisz, Piotr Silmanowicz, Piotr Dobrzyński, Elżbieta Pamuła

**Affiliations:** 1 Department of Biomaterials and Composites, Faculty of Materials Science and Ceramics, AGH—University of Science and Technology, Al. Mickiewicza 30, 30-059 Kraków, Poland; krok@agh.edu.pl (M.K.-B.); kmr@agh.edu.pl (K.R.); lucjarumian@gmail.com (Ł.R.); 2 Department of Cytobiology, Faculty of Pharmacy, Collegium Medicum, Jagiellonian University, ul. Medyczna 9, 30-688 Kraków, Poland; elzbieta.menaszek@uj.edu.pl; 3 Department and Clinic of Animal Surgery, Faculty of Veterinary Medicine, University of Life Sciences, ul. Głęboka 30, 20-612 Lublin, Poland; moski71@gmail.com (M.O.); piotrsil@poczta.onet.pl (P.S.); 4 Department of Electroradiology, Collegium Medicum, Faculty of Health Science, Jagiellonian University, ul. Michałowskiego 12, 31-126 Kraków, Poland; piotr.malisz@uj.edu.pl; 5 Centre of Polymer and Carbon Materials, Polish Academy of Sciences, ul. Curie-Sklodowskiej 34, 41-800 Zabrze, Poland; pdobrzynski@cmpw-pan.edu.pl; 6 Faculty of Science & Technology, Jan Długosz University in Częstochowa, ul. Armii Krajowej 13/15, 42-200 Częstochowa, Poland

**Keywords:** PLGA, scaffolds, collagen, hydroxyapatite, in vivo tests

## Abstract

Poly(l-lactide-*co*-glycolide) (PLGA) porous scaffolds were modified with collagen type I (PLGA/coll) or hydroxyapatite (PLGA/HAp) and implanted in rabbits osteochondral defects to check their biocompatibility and bone tissue regeneration potential. The scaffolds were fabricated using solvent casting/particulate leaching method. Their total porosity was 85% and the pore size was in the range of 250–320 µm. The physico-chemical properties of the scaffolds were evaluated using scanning electron microscopy (SEM), energy dispersive X-ray spectroscopy (EDX), X-ray diffractometry (XRD), X-ray photoelectron spectroscopy (XPS), Fourier transform infrared spectroscopy (FTIR), sessile drop, and compression tests. Three types of the scaffolds (unmodified PLGA, PLGA/coll, and PLGA/HAp) were implanted into the defects created in New Zealand rabbit femoral trochlears; empty defect acted as control. Samples were extracted after 1, 4, 12, and 26 weeks from the implantation, evaluated using micro-computed tomography (µCT), and stained by Masson–Goldner and hematoxylin-eosin. The results showed that the proposed method is suitable for fabrication of highly porous PLGA scaffolds. Effective deposition of both coll and HAp was confirmed on all surfaces of the pores through the entire scaffold volume. In the in vivo model, PLGA and PLGA/HAp scaffolds enhanced tissue ingrowth as shown by histological and morphometric analyses. Bone formation was the highest for PLGA/HAp scaffolds as evidenced by µCT. Neo-tissue formation in the defect site was well correlated with degradation kinetics of the scaffold material. Interestingly, around PLGA/coll extensive inflammation and inhibited tissue healing were detected, presumably due to immunological response of the host towards collagen of bovine origin. To summarize, PLGA scaffolds modified with HAp are the most promising materials for bone tissue regeneration.

## 1. Introduction

Bone tissue has regenerative capacity, but when a critical size defect occurs, regeneration becomes very difficult or even impossible. The regenerative capacity of tissues also decreases with human age [1]. Lack of bone regeneration does not only pose a serious health issue for the patient, but may also induce numerous physiological and economic problems related to long-term treatment [2]. Therefore, novel solutions and biomaterials are being developed to support tissue regeneration. To date, numerous scaffolds based on ceramics (e.g., titanium dioxide [3,4]), metals [5], or natural and synthetic polymers [6,7]) have been developed and evaluated in terms of their biocompatibility and bioactivity. 

Three dimensional, highly porous scaffolds based on resorbable polymers, such as poly(l-lactide-*co*-glycolide) (PLGA), are widely considered as bone tissue engineering (BTE) substrates. PLGA gives opportunity to produce implants with different microstructure, porosity (including variable pore size) and mechanical properties. The porosity and interconnectivity of pores are important factors influencing cell infiltration, migration, vascularization, nutrient and oxygen flow, or removal of waste [8,9]. Several techniques such as porogen leaching, fiber bonding, electrospinning, 3D printing, or phase separation/freeze-drying have been developed to obtain scaffolds for BTE [10]. 

PLGA is approved for use in humans by the Food and Drug Administration (FDA) and the European Medicines Agency (EMA). PLGA main advantage is that in the presence of water in body fluids, it undergoes hydrolytic degradation to lactic and glycolic acids, that are further metabolized in the Krebs cycle, leaving no possible toxic degradation products [11]. PLGA degradation kinetics can be controlled upon adjusting of lactide to glycolide unit ratio, chain structure, molecular weight, or crystallinity [12]. 

PLGA is usually synthesized by ring-opening polymerization with the use of tin compounds as polymerization initiator [13], but literature related to its synthesis with the use of low-toxic initiators is scarce [14]. The FDA approved tin compounds as food additives, but it is questionable to use them in medical implants [15,16]. Especially, organic tin compound tin(II) 2-ethylhexanoate (Sn(Oct)_2_), which is commonly used as ring-opening polymerization initiator, provokes adverse effects in the human body as it may accumulate in tissues and organs [17]. We think that the use of non-toxic initiators is not yet fully explored approach. 

Our team has been studying PLGA synthetized with the use of zirconium(IV) acetylacetonate, which was found more cytocompatible than PLGA produced with Sn(Oct)_2_ [14,18,19]. We found that PLGA 85:15 (i.e., with 85:15 molar ratio of l-lactide to glycolide) degradation time depends on the form of the processed material: PLGA foils degraded much faster than PLGA porous scaffolds, both in vitro (in PBS solution) and in vivo (implantation in muscle tissue in rats) [20]. In our more recent study, we showed that PLGA modification with hydroxyapatite (HAp) deposited by biomimetic approach is beneficial for osteoblast adhesion, infiltration, and proliferation in the entire volume of porous scaffold [21]. Moreover, we found that modification of the PLGA scaffolds with artificial extracellular matrices (ECM) containing collagen type I and sulfated hyaluronic acid is beneficial for early and late osteogenic differentiation and mineralization of human mesenchymal stem cells (hMSC) [22], thus promising for BTE. To go a step further with possible translation, the scaffolds should be tested in a model more relevant to clinical application.

Thus, in this study, we wanted to confirm biocompatibility of our PLGA scaffolds in an in vivo osteochondral tissue rabbit model. In addition to scaffold modification with HAp we deposited collagen type I, i.e., the most abundant protein in bone tissue ECM. 

The aim of this study was to: (i) Modify the PLGA scaffolds with hydroxyapatite (HAp) or collagen type I, (ii) to find out if applied modifications are beneficial for the healing process in critical-size osteochondral defects in rabbits and (iii) to evaluate in vivo degradation of PLGA and the influence of HAp/coll surface modifications on degradation rate. 

## 2. Results

**Figure 1:**
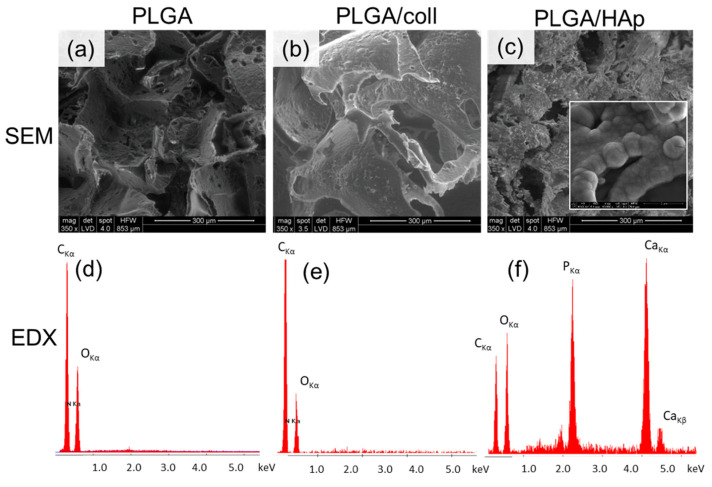
Microstructure by scanning electron microscopy (SEM) (**a**–**c**) and elemental analysis by energy dispersive X-ray spectroscopy (EDX) (**d**–**f**) of the scaffolds: Poly(l-lactide-*co*-glycolide) (PLGA) (**a**,**d**), Poly(l-lactide-*co*-glycolide) modified with collagen type I (PLGA/coll) (**b**,**e**), and Poly(l-lactide-*co*-glycolide) modified with hydroxyapatite (PLGA/HAp) (**c**,**f**). Insert in PLGA/HAp (**c**) shows globular cauliflower mineral deposits typical for low-crystalline hydroxyapatite.

Fabricated scaffolds had interconnected pores (Figure 1a–c). The average pore size was between 250 and 320 µm, thus similar to the size of NaCl grains used as porogens. Scaffold porosity of 85% corresponded to volume faction of porogen used in the manufacturing step as shown in our earlier work [22]. Modification with collagen did not significantly influence morphology and porosity of the scaffolds. 

**Figure 2:**
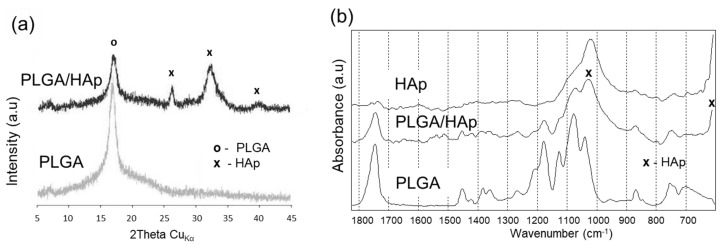
X-ray diffractometry (XRD) patterns (**a**) and Fourier transform infrared (FTIR) spectra (**b**) of PLGA and PLGA/HAp scaffolds. Pics (**a**) and bands (**b**) marked by “x” are characteristic for hydroxyapatite.

The amount of collagen deposited on PLGA/coll surface was too low to be detected by energy dispersive X-ray spectroscopy (EDX) (Figure 1e), and carbon and oxygen contents were the same as for PLGA (Figure 1d). However, X-ray photoelectron spectroscopy (XPS) analysis of PLGA/coll (Table 1) revealed 2.8 at.% of nitrogen presence on the surface, i.e., the marker of all proteins. The deposition of HAp on PLGA drastically changed scaffold microstructure, as pore surface became rougher. At higher magnification (Figure 1c, insert) cauliflower-like structures, typical for low-crystalline hydroxyapatite, can be observed [23]. The presence of HAp was also confirmed by EDX (Figure 1f) and XPS (Table 1) analyses. The Ca/P atomic ratio was close to 1.66, thus characteristic for hydroxyapatite. The crystalline structure of HAp was also evidenced by X-ray diffractometry (XRD) (Figure 2a), as peaks characteristic for HAp appeared at 2θ around 26° (corresponding to 002 plane), 32° (211 plane), and 40° (310 plane) [23,24]. Fourier transform infrared (FTIR) spectra (Figure 2b) also confirmed the presence of HAp coating on PLGA scaffold (intensive bands originating from PO_4_^3−^ group at 600 cm^−1^ and at 1000–1100 cm^−1^) [25]. The bands corresponding to PLGA were still present in the spectra for PLGA/HAp scaffold (1757 cm^−1^ attributed to C=O stretching or 1090 cm^−1^ and 1185 cm^−1^ originating from C-O stretching) [26].

Modification with collagen or HAp increased scaffold wettability (Table 1), as the water contact angle dropped from 126.7 ± 9.3° for non-modified PLGA to 103.4 ± 6.8° for PLGA/coll. Water contact angle for PLGA/HAp was impossible to be determined because the water droplets immediately penetrated the scaffolds, showing that the scaffold was super-hydrophilic. Contrary to collagen modification, the presence of HAp on PLGA surface significantly increased mechanical properties of PLGA scaffolds—both compressive strength and E modulus were improved in PLGA/HAp. 

**Figure 3:**
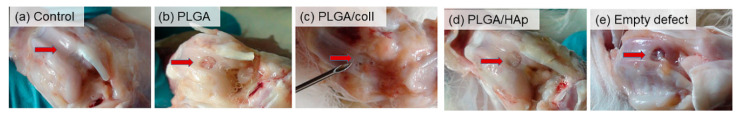
Rabbit femoral trochlear of control knee (**a**) and four weeks post-surgery when defect was filled with PLGA scaffold (**b**), PLGA/coll scaffold (**c**), PLGA/HAp (**d**), and when the defect was left empty (**e**). Arrows show trochlear groove (**a**), defects filled with scaffolds (**b**–**d**), and empty defect (**e**).

PLGA reference scaffolds and those modified with collagen or hydroxyapatite were implanted into osteochondral defects of New Zealand rabbits to evaluate their biocompatibility and bone regenerative potential. Rabbit femoral trochlear of control knee is shown in Figure 3a. An empty defect was used as a reference. Four weeks post-implantation the most advanced healing was observed for PLGA/HAp and PLGA scaffolds (Figure 3b,d, respectively), while for PLGA/coll scaffolds (Figure 3c) inflammation, manifested by an extensive exudate in the synovial cavity, was observed. The healing was retarded when the defect was not filled with any scaffold (Figure 3e). 

**Figure 4:**
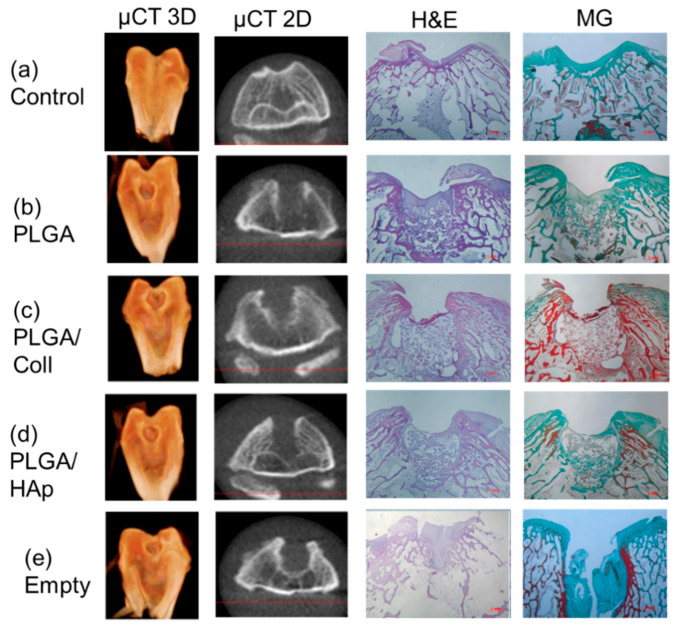
Computer tomography in 3D and 2D projection (first and second column) and histological evaluation after hematoxylin and eosin (third column) and Masson-Goldner staining (fourth column) 4 weeks post-implantation: (**a**)—control, (**b**)—defect filled with PLGA scaffold, (**c**)—defect filled with PLGA/coll scaffold, (**d**)—defect filled with PLGA/HAp scaffold, (**e**)—empty defect.

Visual observations were confirmed by micro-computed tomography (µCT) examination and histological staining (Figure 4). Four weeks after implantation, none of the defects were fully regenerated. Hyaline cartilage was also not healed and the defects were filled with unmineralized tissue. Empty defect was filled with fibrous tissue as shown by Masson–Goldner staining. However, in the case of PLGA and PLGA/HAp scaffolds, the defects were smaller in comparison to PLGA/coll or control group as proved by µCT. The ingrowth of a new tissue observed in PLGA/HAp scaffolds was initially observed at the peripheries of the defect, continuing towards the center of the defect. 

**Figure 5:**
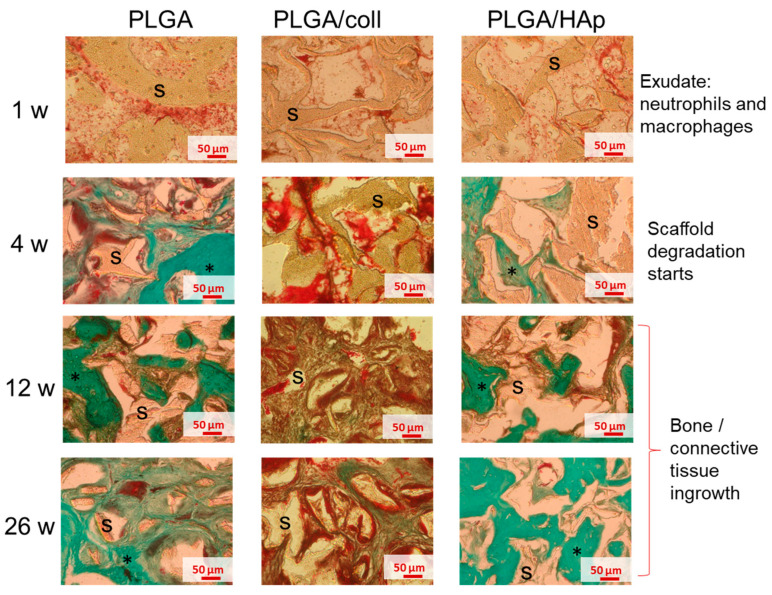
Histological analysis after Masson–Goldner staining of PLGA (first column), PLGA/coll (second column), and PLGA/HAp (third column) scaffolds at the implantation site after 1 week (first panel), 4 weeks (second panel), 12 weeks (third panel), and 26 weeks (fourth panel) post-implantation; s—scaffold, star (*)—tissue ingrowth. The pictures were taken in the central part of the defect filled with the scaffold.

Histological examination of the samples showed that at one-week post-implantation, the scaffolds were filled with exudate containing inflammatory cells: Mainly neutrophils and some macrophages (Figure 5, first panel). Inflammation intensity at this time period was the same for all analyzed scaffolds. Four weeks post-implantation degradation of the scaffolds was visible in all the samples (Figure 5, second panel). Twelve and 26 weeks post-implantation, tissue ingrowth was observed predominantly in the case of PLGA and PLGA/HAp scaffolds (Figure 5, third and fourth panels, respectively). In PLGA/coll scaffolds, inflammatory cells were still visible and tissue ingrowth was retarded. 

**Figure 6:**
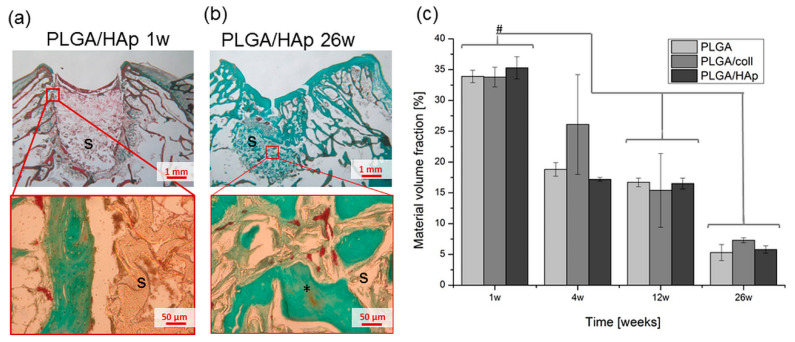
Histological analysis after Masson–Goldner staining of PLGA/HAp scaffold 1 week post-implantation (**a**) and PLGA/HAp scaffold 26 weeks post-implantation (**b**); s—scaffold, star (*)—tissue ingrowth. Material volume fraction of PLGA, PLGA/coll and PLGA/HAp scaffolds at the implantation site after 1, 4, 12 and 26 weeks post-implantation (**c**); statistically significant differences at ^#^ *p* < 0.05 according to ANOVA.

Histological analysis of the scaffold implantation sites performed for different periods of time, i.e., from week 1 until week 26 from surgery, allowed us to assess scaffolds degradation in vivo. Interestingly, degradation of all types of scaffolds proceeded in a similar manner (Figure 6). One week after implantation, tissue defects were filled with the scaffold material, but there was a gap between the tissue and the scaffold (Figure 6a). For longer time from the surgery, the tissue defects were infiltrated with cells and the neo-tissue was created on the basis of the degrading scaffold. Twenty-six weeks after implantation, the amount of scaffold material was significantly reduced, and the remnants of the scaffolds were tightly embedded in the new tissue (Figure 6b). 

Morphometric analysis (Figure 6c) confirmed that in all cases, the volume fraction of the material observed in histological sections gradually decreased from above 30% (1 week after the implantation) to around 5% (26 weeks after the implantation). No significant differences in degradation rate were observed between PLGA, PLGA/coll, and PLGA/HAp scaffolds after 12 and 26 weeks from implantation indicating the collagen or HAp modification did not influence in vivo degradation of the material.

**Figure 7:**
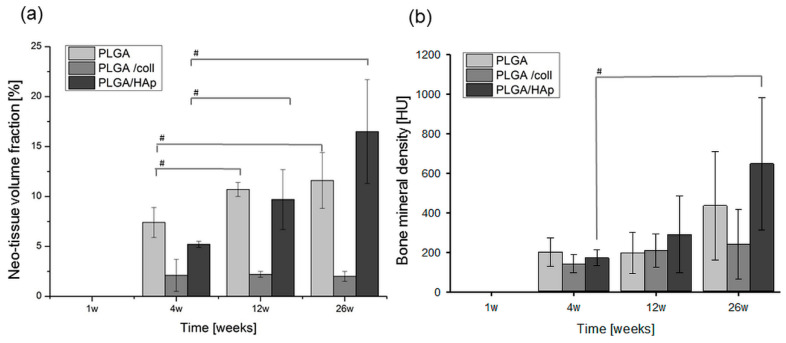
Neo-tissue volume fraction assessed by morphometric analysis of histological pictures (**a**) and bone mineral density measured by µCT (**b**) at the implantation site of PLGA, PLGA/coll, and PLGA/Hap scaffolds after 1, 4, 12, and 26 weeks post-implantation; statistically significant differences at ^#^ *p* < 0.05 according to ANOVA.

Volume fraction of novel tissue created within the scaffolds and on the base of the degraded scaffolds was quantified using morphometric analysis of histological pictures (Figure 7a). The results confirmed that PLGA and PLGA/HAp scaffolds stimulated tissue regeneration and the amount of tissue formed within those materials increased in the course of time. After 26 weeks post-implantation, the highest tissue volume fraction was present in defects filled with PLGA/HAp scaffolds. In the case of PLGA/coll scaffolds, the amount of tissue was not increasing. Mineralization of novel tissue created on the base of the scaffolds as studied by µCT (Figure 7b) showed that bone mineral density tended to increase as a function of time. The most pronounced bone mineral density and thus bone tissue regeneration was observed in the case of PLGA/HAp scaffolds. 

## 3. Discussion

Solvent casting/particulate leaching method was proved suitable for fabrication of highly porous PLGA scaffolds. This technique allows for fabrication of scaffolds based on different polymers (e.g., polylactide or polycaprolactone) and with strictly defined morphologies [27]. In comparison to other scaffold fabrication techniques (e.g., 3D printing or electrospinning), it is relatively simple and inexpensive. In our study, NaCl was used as a porogen. SEM analyses revealed that the pore size in the scaffolds was determined by the size of NaCl grains used for scaffold fabrication [21,22,28]. What is more, the pores were interconnected, which is particularly beneficial for tissue regeneration purposes, as after the implantation, body fluids, nutrients, and cells will be able to penetrate the whole volume of the scaffold. Proper pore interconnections also allow cell migration and interaction with other cells and ECM molecules [29].

Collagen type I or hydroxyapatite have been used in order to improve resorbable scaffold biocompatibility and promote bone tissue regeneration. Both collagen type I and HAp are well known for their osteogenic potential [30,31,32]. It was already evidenced by Lee et al. [33], that the adhesion of hMSC was significantly improved upon coating of polylactide surface with collagen. HAp, being similar to the one present in native bone tissue, provides structural support and enhances osteoblast adhesion [34]. Due to high porosity of the scaffolds and well developed interconnection between pores, applied modifications were deposited throughout the whole volume of the scaffolds, not just on their surface. HAp coating significantly increased scaffold wettability, which is crucial in terms of osteoblast adhesion and in vivo implantation. Only upon adsorption of water molecules, ions, and proteins originating from body fluids can the cells attach to the surface of the implanted material, spread on its surface, proliferate, and further form regenerated tissue [35]. 

Our previous studies on HAp modified PLGA proved that the proposed method of modification allows for obtaining modified materials, and the tests confirmed the presence of HAp not only on the external surface, but also on the pore walls inside the scaffold. The results of the cell vitality tests suggest that the PLGA scaffolds mineralized by immersion in SBF are biocompatible for osteoblasts and HAp modification improves cell adhesion to the scaffold walls [21,36]. 

In this study, upon implantation of PLGA-based scaffolds in osteochondral defects in the knees of experimental rabbits, some differences were observed between PLGA, PLGA/coll, and PLGA/HAp scaffolds. Through the whole experiment, PLGA/coll scaffolds showed increased neutrophil and macrophage infiltration and intensive inflammatory response. This observation was particularly surprising for us, as in general collagen is regarded as biocompatible and low-immunogenic material [37]. This evidenced that xenograft proteins (in our case collagen obtained from bovine tendons) evoked more pronounced immunological response in rabbits in comparison to synthetic PLGA or biomimetic HAp. The helical part of collagen macromolecule is relatively stable and similar in different animal species, however amino acid composition of its terminal, non-helical parts vary to a greater extent [38]. Such a major antigenic reaction related to terminal parts of a protein was observed by Pontz et al. [39] in rabbit antiserum upon exposure to solubilized bovine collagen. A similar reaction was discovered in rabbit, while rat was used as a collagen donor [40]. Besides the interspecies difference, collagen is a material of natural origin, thus its properties may vary depending on production batch, purification procedure, or storage conditions [41,42,43]. Several concerns over the use of collagen of bovine origin have already been raised, mainly connected to possible transmission of bovine spongiform encephalopathy (BSE) or viruses [37,44]. Thus, novel sources of collagen are being developed, including fish skin, jelly fish, plants, or synthetic KOD (a synthetic analogue of collagen composed of 36 amino acids organized into triple-helixes) [45,46]. 

Contrarily to PLGA/coll, both PLGA and PLGA/HAp scaffolds were more effective in supporting bone tissue regeneration, as no inflammatory responses manifested by extensive exudate were observed for longer observation periods. In the course of time, new connective tissue was formed within scaffold pores. The ingrowth of the tissue was initially observed in scaffold peripheries, so close to native tissue, however 26 weeks after implantation, newly formed tissue was also found in the whole volume of the defect filled with degrading scaffolds. 

Regardless of the presence or type of scaffold modification, PLGA degraded gradually throughout the experiment, leaving more space for tissue ingrowth and regeneration. No significant differences were observed between PLGA, PLGA/coll, and PLGA/HAp in terms of degradation kinetics. PLGA 85:15 was selected for scaffold manufacturing as it was already evidenced that its in vivo degradation is similar to the degradation in vitro, and it is thus more predictable [20]. On the contrary, PLGA 50:50 degrades significantly faster, due to its chemical chain structure and also leads to more pronounced acidification of the surrounding tissues due to autocatalytic effect [47]. 

Although initially both PLGA and PLGA/HAp scaffolds showed similar behavior, by the end of the experiment, the volume of regenerated tissue and its mineralization toward matured bone tissue was higher in the case of PLGA/HAp indicating simulative potential of HAp coating. 

Thanks to the stimulation of bone defect healing as proved in this study, PLGA scaffolds modified with biomimetic HAp are promising materials in the field of bone tissue regeneration. In addition, they can be further modified to provide them with antibacterial properties or other biologically active molecules. Different antibacterial drugs preventing biofilm development or moieties stimulating cell differentiation and tissue regeneration can be incorporated into HAp coating during deposition process. Since the PLGA scaffolds were modified with HAp deposited from SFB solution, no high temperature treatment was necessary. Therefore, the modification of HAp coating with temperature labile substances, such as peptides, proteins, or polymeric nanocarriers, should be possible. 

Tian et al. [48] prepared HAp coating loaded with silver nanoparticles. The excellent antibacterial activity of such materials was attributed to the dissolution of silver nanoparticles and release of silver ions leading to disruption of bacterial replication, and in consequence bacterial death. Gronowicz et al. [49] deposited bone morphogenetic protein-2 (BMP-2) on the surface of a synthetic bone graft, covered the scaffold’s surface with biomimetic calcium phosphate (bCaP), and finally functionalized its surface with fibroblast growth factor-2 (FGF-2). As demonstrated in vivo, bCaP coating delayed initial exposure of the cells to BMP-2, while FGF-2 stimulated cell adhesion. Further exposure to BMP-2 significantly improved cell differentiation and tissue regeneration. 

To sum up, the following research was aimed at in vivo evaluation of highly porous scaffolds made of PLGA synthesized with the use of non-toxic zirconium compound. The scaffolds were modified with collagen type I of bovine origin or biomimetic HAp in order to improve their biocompatibility and induce bone tissue regeneration. It was evidenced, that PLGA and PLGA/HAp scaffolds augmented bone tissue regeneration in the rabbit model. Surprisingly, PLGA/coll scaffolds were not effective, as the presence of xenotypic collagen induced inflammatory reaction, which hampered neo-tissue formation. The developed PLGA/HAp material is promising as bone tissue engineering scaffolds that can be further refined e.g., via encapsulation of growth factors or other biologically active moieties.

## 4. Materials and Methods 

### 4.1. Chemicals and Polymer Synthesis, Scaffolds Manufacturing and Modification

Poly(l-lactide-co-glycolide) (PLGA 85:15; Mn = 100 kDa, d = 2.1) was synthesized by open-ring polymerization with nontoxic initiator zirconium(IV) acetylacetonate [19]. The scaffolds were made by solvent casting/particulate leaching process, details were described previously [21,36]. In brief, 10% *w*/*v* PLGA were dissolved in dichloromethane (DCM, Sigma-Aldrich, Darmstadt, Germany) and mixed with porogen NaCl (Sigma-Aldrich, Darmstadt, Germany) with a defined size of 250–320 µm (salt/PLGA volume fraction 85%). In order to shape it, the obtained paste was placed in syringes (4 mm in diameter) and left for 24 h to evaporate the solvent. After that, the salt/PLGA composites were cut into slices (5 mm in height) and immersed in UHQ-water to leach out the porogen. Water was replaced several times until water conductivity was below 2 µS/cm and all salt was released; it usually took 5 days. 

For scaffold modification, the simulated body fluid (SBF) for biomimetic deposition of HAp and collagen solution (40 µg/mL, type I, from calf skin, C819, aseptically prepared, suitable for cell culture, Sigma-Aldrich, Darmstadt, Germany) were used. The deposition process lasted 12 days and 24 h for HAp [36] and collagen [50], respectively.

### 4.2. Scaffold Characterization

The scaffolds have been characterized in terms of microstructure, wettability, chemical composition, and in vivo properties. 

Scanning electron microscope (SEM, Nova, NanoSEM, FEI, Hillsboro, OR, USA) was used for microstructure and surface morphology characterization. Before analysis, the samples were coated with a thin carbon layer to make them conductible. X-ray (EDX) spectroscopy (Link AN 10000, Oxford Instruments, High Wycombe, UK), was used for analysis of elemental composition. 

The structure of phosphate deposits and PLGA was investigated by X-ray powder diffraction (XRD) (Empyrean. PANalytical B.V., Almelo, The Netherlands). Prior to XRD analyses, the scaffolds were crushed into powders. The samples were also evaluated by Fourier transform infrared (FTIR) spectroscopy using FTS-60V Digilab Division spectrometer from BIORAD (Hercules, CA, USA). Transmission spectra were recorded with a resolution of 4 cm^−1^ by averaging of 256 scans for each spectrum. Before analysis, the scaffolds were crushed and mixed with KBr to form pellets. 

The XPS spectra were recorded using an SSI X-Probe (SSX-100/206) spectrometer from Surface Science Instruments (Mountain View, CA, USA). Briefly, monochromatized AlKα X-ray radiation (1486.6 eV) was used. Charge stabilization was achieved using an electron flood gun set at 6 eV and placing a grounded nickel grid 3 mm above the sample surface. The pressure during the analyses was between 4.0 × 10^−9^ and 2.0 × 10^−9^ Torr. The irradiated zone was an elliptic spot with a longer axis of 1000 µm. The constant pass energy was 150 and 50 eV for wide-scan and detailed peak analysis, respectively. The angle between the normal to the sample surface and the direction of the photoelectron collection was 55°. The following sequences of spectra were recorded: Wide-scan spectrum, C1s, O1s, N1s, Ca2p, P2p, and C1s again to check for the absence of sample degradation. No modification of the C1s peak shape under X-ray irradiation was noticed, indicating that the samples did not undergo degradation during analysis. The data treatment was performed with the ESCA 8.3 D software provided by the spectrometer manufacturer. The binding energy (EB) of the main lines (C1s, O1s, N1s, Ca2p, P2p) was determined by setting the value of 284.8 eV for the main C1s component, due to carbon only bound to carbon and hydrogen. The peak area was determined using Shirley-type nonlinear background subtraction. Intensity ratios were converted into molar concentration ratios using the sensitivity factors proposed by the manufacturer (Scoffield emission cross sections, variation of the electron mean free path ac-cording to the 0.7 power of kinetic energy, constant transmission function).

Water contact angle of the scaffolds was evaluated by DSA10Mk2 (Krüss, Hamburg, Germany) from a drop shape analysis based on 12 individual measurements using ultra-high quality water (UHQ-water) droplets (0.5 mL). The results were presented as mean ± SD (standard deviation).

The compressive strength and compressive E modulus of the scaffolds were measured using ZWICK 1435 universal testing machine (Zwick Roell, UIm, Germany) at a cross head speed of 2 mm/min. The determination of E modulus was based on the slope in the initial elastic portion of the stress–strain diagram. The results were presented as mean ± SD for 6 individual scaffolds for each scaffold type. The results for PLGA/coll and PLGA/Hap were found statistically different from control PLGA scaffold if * *p* < 0.05 according to *t*-test. 

### 4.3. In Vivo Evaluation in a New Zealand Rabbit Model

In vivo evaluation of the scaffolds was performed in a New Zealand rabbit model, according to ISO 10993-6 standard and a protocol approved by the Local Ethic Committee of the University of Life Sciences, Lublin, Poland (No 43/2008, approved 01/07/2008). Three types of the scaffolds (i.e., PLGA, PLGA/HAp, PLGA/coll), sterilized with oxygen peroxide plasma (Sterrad 120, ASP, J&J, New Brunswick, NJ, USA), were implanted in the defects created in rabbit femoral trochlears. For anesthesia, a mixture of xylazine and ketamine was administered by intramuscular injection (5 mg/kg xylazine and 35 mg/kg ketamine). The implantation time was 1, 4, 12, and 26 weeks. For each biomaterial and implantation period, 3 rabbits were allocated and each received 2 materials or the tibial tuberosity defect remained empty, and one rabbit acted as control, so in total, 25 animals were used. The animals were monitored throughout the study. 

**Figure 8:**
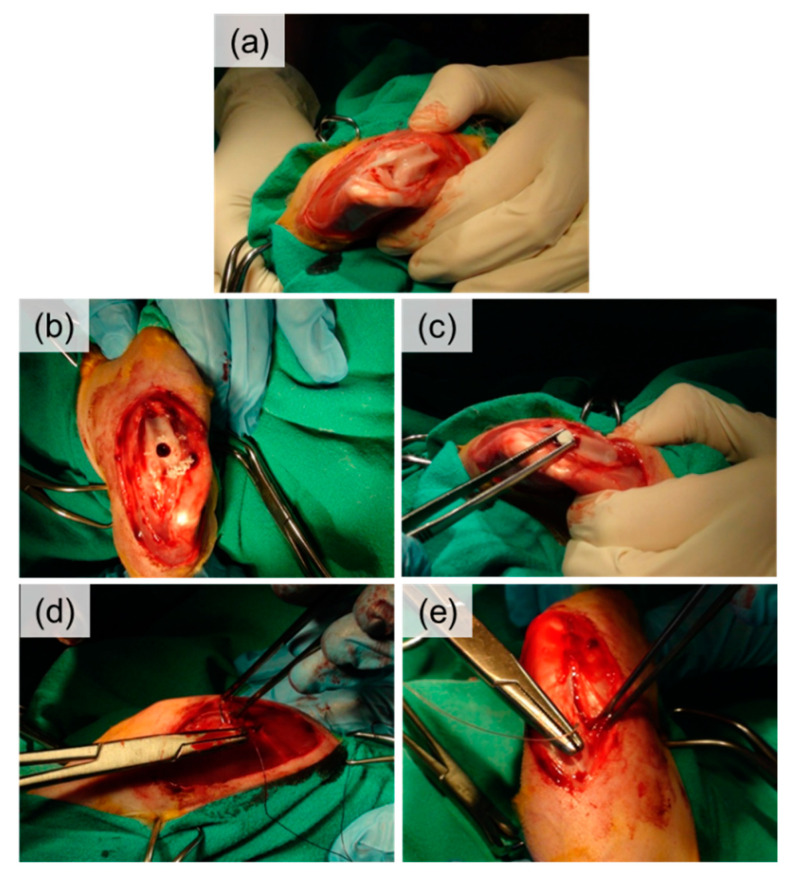
Surgical procedure of scaffold implantation into rabbit femoral trochlear: (**a**)—exposed surface of femoral trochlear, (**b**)—hole drilled in the trochlear groove, (**c**)—scaffold introduction, (**d**)—tissues apposed with absorbable sutures, (**e**)—skin apposed with non-absorbable sutures.

Before the surgery the area surrounding the knee joint was shaved and a venflon was placed into the marginal ear vein (*v. auricularis marginalis*). The state of sedation and analgesia was achieved (after nearly 10 min), and to maintain the anesthesia, ketamine was used as a continuous infusion using an infusion pump at a dose of 0.5 mg/kg/min iv. The incision was made on the lateral side of the stifle joint. It extended from the lateral distal end of the femur to approximately 2 cm below the tibial tuberosity. Next, the incision fascia lata, laterally on the block of the knee was done, straight along the patellar ligament. At the end, the capsule of the knee was cut. The patella with vastus lateralis muscle was moved medially, showing the surface of the femoral trochlear (Figure 8a). The round hole with a diameter of 4 mm and depth of 5 mm was drilled in the middle of the trochlear groove of the femur (Figure 8b). The scaffolds were introduced in the prepared defects (Figure 8c). Absorbable sutures (Polyglactine 910, 3/0) were used for apposed internal tissues, while nonabsorbable suture material (Polyamide, 3/0) was used on the apposed skin (Figure 8d,e).

To alleviate postoperative pain, rabbits were given an anti-pain drug (butorphanol at a dose of 0.1 mg/kg) and an anti-inflammatory drug (Sul-Tridin 24% at a dose of 30 mg/kg once a day for five days). After recovery rabbits were able to freely move in the cages, and most of the animals revealed no signs of knee dysfunction. After 10 days, skin sutures were removed. Before the end of the study, one rabbit in the PLGA/coll group showed a significant swelling of the knee sustained in a week after surgery. The animal had limited range of motion and showed a first-degree lameness. After the euthanasia, it was determined that the joint had inflammation, an increased amount of synovial fluid, and degenerative changes. These causes were the effect of the clinical changes described in the functioning of the joint. After each time point, the rabbits were sacrificed. Rabbits were introduced into a state of general anesthesia with xylazine and ketamine. Euthanasia was properly performed by intracardiac injection of sodium pentobarbital, after reaching deep anesthesia. After euthanasia, explant was collected and immersed in 4% formalin for further testing. 

Micro-computed tomography (µCT) evaluation of the explants was done using GXCB-500/i-CAT (Gendex Dental System, Cusano, Italy) volumetric tomography. An isometric voxel size was set to 125 × 125 × 125 µm^3^, total scanning time was 23 s. iCAT Vision software in DICOM standard was used for data analyses. 3D and 2D projections of the rabbit knee were obtained. To determine bone mineral density (BMD), the area of 7.2 mm^2^, corresponding to the center of the defect, was analyzed and expressed in Hounsfield units (HU). 

For histological staining, the explants were washed from formalin using tap water for 24 h. The samples were decalcified using hydrochloric acid for 5 days (TBD-1 Rapid decalcifier, Thermo Shandon Ltd, UK). Decalcified samples were immersed in graded ethanol series (50%, 70%. 90%, 96%, and twice in 100%) for 1 day each, and finally washed twice in xylene for 1 h. In the end, treated explants were immersed in liquid paraffin (Histoplast) for 3 days at 55 °C. As-prepared paraffin blocks were cut into 9 µm sections using a rotary microtome (RM 2145, Leica Microsystems, Wetzlar, Germany). Tissue slices were deparaffinized using xylene and rehydrated in ethanol series. The specimens were stained with Masson–Goldner trichrome staining and hematoxylin and eosin staining in accordance to standard protocols. 

Morphometric analyses on Masson–Goldner stained samples were done using stereoscopic microscope under 10x magnification (Zeiss SteREO Discovery V8, Zeiss, Oberkochen, Germany). Each image was divided with grid into 100 square sections (100 µm × 100 µm) using Cell^D software (Electro Optics, Olympus, Cambridge, UK). The formation of neo-tissue and scaffold degradation were evaluated based on counting of grid intersections laying in regions identified as neo-tissue or scaffold. The percentage volume fraction of either neo-tissue or scaffold was determined as:

(1) $$Neo-tissue\;or\;scaffold\;volume\;fraction\;\left[\%\right]=\frac{{\mathrm{Intersections}}_{A}}{{\mathrm{Intersections}}_{\mathrm{total}}}*100\%$$

where Intersections_A_ is the number of intersections laying in regions identified as neo-tissue or scaffold and Intersections_total_ is the total number of intersections in the image. For each sample group at least 3 representative images were analyzed using 6–9 grid placements. 

The statistical data were treated using a one-way analysis of variance (one-way ANOVA) followed by Tuckey’s post-hoc test. The differences between the samples were considered significant if *p* < 0.05. The assumptions of normal distribution and equal variance were verified using the Shapiro–Wilk and Levene median test, respectively (*p*-value > 0.05). The analyses were performed using SigmaPlot 12.3 software (Systat Software, Inc., San Jose, CA, USA). The results are presented as mean ± standard deviation (SD).

## 5. Conclusions

This research was focused on the development of PLGA scaffolds for bone tissue engineering. The scaffolds were highly porous (85% porosity) and had well developed, interconnected pores of 250–320 µm in diameter. PLGA scaffolds were modified with bovine collagen type I or biomimetic HAp. The biocompatibility of PLGA, PLGA/coll and PLGA/HAp scaffolds was evaluated in vivo in a rabbit model. Both PLGA and PLGA/HAp scaffolds improved tissue ingrowth and regeneration. On the contrary, PLGA/coll scaffolds induced considerable inflammatory reaction presumably due to xenogeneic origin of collagen. The degradation of all scaffolds was similar and progressed in a uniform manner in the course of time. The developed materials can be further optimized (e.g., via decoration with biologically active compounds) to enhance bone tissue regeneration. 

## Figures and Tables

**Table 1:** Properties of the scaffolds: surface chemical composition (excluding hydrogen), water contact angle, strength, and modulus E in compression test.

Scaffold Type	Surface Chemical Composition (at.%)					Water Contact Angle (Deg.)	Strength σ (MPa)	Modulus E (MPa)
	C	O	Ca	P	N			
PLGA	61.0	39.0	^bdl^	^bdl^	^bdl^	126.7 ± 9.3	0.58 ± 0.10	0.63 ± 0.12
PLGA/coll	56.0	40.2	^bdl^	^bdl^	2.8	103.4 ± 6.8 *	0.61 ± 0.09	0.66 ± 0.15
PLGA/HaAp	17.0	66	10.6	6.4	^bdl^	^nd^	1.14 ± 0.31 *	1.72 ± 0.37 *

^bdl^—below detection limit, ^nd^—not determined, *—statistically different from control PLGA scaffold according to *t*-test, *p* < 0.05.

## Abbreviations

bCaP	Biomimetic calcium phosphate
BMD	Bone mineral density
BMP-2	Bone morphogenetic protein-2
BTE	Bone tissue engineering
coll	Collagen type I
DCM	Dichloromethane
ECM	Extracellular matrix
EDX	X-ray spectroscopy
EMA	European Medicines Agency
FDA	Food and Drug Administration
FGF-2	Fibroblast growth factor-2
FTIR	Fourier transform infrared spectroscopy
HAp	Hydroxyapatite
HU	Hounsfield units
µCT	Micro-computed tomography
PLGA	Poly(l-lactide-*co*-glycolide)
SBF	Simulated body fluid
SEM	Scanning electron microscopy
XPS	X-ray photoelectron spectroscopy
XRD	X-ray powder diffractometry
